# Cavernostomy for Pulmonary Aspergillosis Associated with Destroyed Lung after Surgery for Lung Cancer: Report of 3 Cases

**DOI:** 10.1155/2015/614795

**Published:** 2015-10-20

**Authors:** Ryo Takahashi, Taiki Fujiwara, Hisami Yamakawa

**Affiliations:** ^1^Department of General Thoracic Surgery, National Hospital Organization Chiba-East Hospital, Chiba 260-0856, Japan; ^2^Department of General Thoracic Surgery, Graduate School of Medicine, Chiba University, Chiba 260-0801, Japan; ^3^Department of Surgery, Jinken Clinic, Kanagawa 243-0432, Japan

## Abstract

Slow, progressive, and destructive changes in the residual lung after surgery for lung cancer, known as “destroyed lung,” are delayed nonrecurrent complications. Destroyed lung can be a difficult condition to treat due to repeated infections and is therefore a complication that should not be ignored. We had three cases of intractable pulmonary aspergillosis difficult to treat associated with destroyed lung, after lung cancer surgery. Two of these patients followed a characteristic clinical course, which started with a cystic change just below the pleura and subsequently led to respiratory failure and death due to repeated infections. The third patient followed a similar clinical course and is currently under regular follow-up. Our cases suggest that concomitant occurrence of severe complications following surgery for lung cancer, such as destroyed lung and pulmonary aspergillosis, should be monitored because these complications can lead to respiratory failure and fatal clinical course. Radical surgery is not possible, especially when medical treatment is ineffective in controlling repeated infections and the patient's general condition is worsened due to prolonged chronic inflammation. Therefore, aggressive surgical intervention should be considered before patients worsen.

## 1. Introduction

Pulmonary aspergillosis is a disease caused by infection and saprophytic growth of* Aspergillus* sp. with the formation of a fungus ball in a preexisting lung cavity. The underlying diseases of pulmonary aspergillosis include pulmonary tuberculosis, which is most common, followed by lung cancer, bronchiectasis, and lung abscess [1–3]. Aggressive therapy is required when treating pulmonary aspergillosis, since symptoms including bloody sputum and hemoptysis may appear as a disease progresses; however, it is difficult to completely destroy the fungus ball by antifungal drugs alone. According to the guidelines, surgical resection of the lung is recommended as the first choice of treatment [4]. However, patients with pulmonary aspergillosis are often ineligible for lung resection due to impaired pulmonary function and poor nutrition caused by underlying disease. In such patients, cavernostomy or cavernoplasty is performed to remove the fungus ball [2, 5], based on the notion that mechanical friction associated with the movement of the mass and toxin produced by the fungus are involved in the breakdown of the vessel walls and resulting hemoptysis [6]. We report our experience with three cases of pulmonary aspergillosis that presented with hemoptysis and repeated infections in the residual lung after resection for lung cancer that were treated with cavernostomy. All three patients had surgery alone as a previous treatment for lung cancer with no combined chemoradiotherapy.

## 2. Case Presentations

### 2.1. Case 1 (A 73-Year-Old Male)

*Past History*. Sleeve right upper lobectomy with lymph node dissection for right lung cancer at the age of 67 years. 

*Pathological Findings*. Moderately differentiated epidermoid carcinoma, p-T2bN1M0.

*Pulmonary Function Test Results*. FVC: 2.66 L, %FVC: 74%, FEV_1.0_: 1.44 L, and FEV_1.0%_: 70.6%.

**Figure 1:**
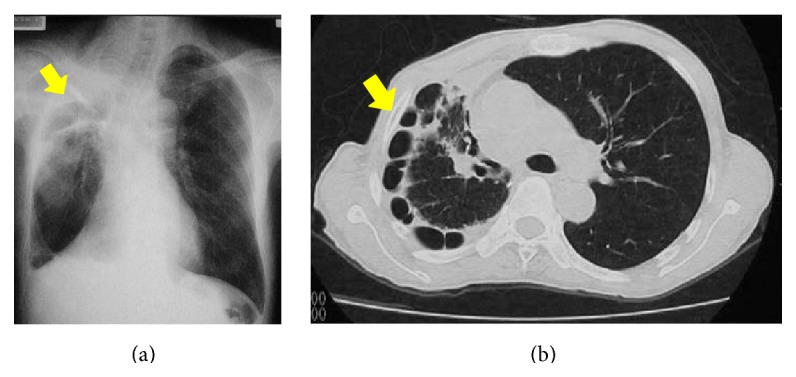
Chest X-ray (a) and chest contrast tomography scan (b) in Case 1. There are interstitial shadows and multiple cysts immediately below the pleura due to shrinkage of the lung, suggesting advanced destroyed lung.

*Clinical Course*. Starting approximately 4 years after his lobectomy, the patient presented with repeated fever, which was relieved by oral antibiotics. Chest CT showed a mild cystic change just below the pleura and his sputum and blood cultures revealed no significant bacteria. At 5 years 8 months after his lung resection, the patient developed bloody sputum and his chest CT showed advanced destroyed lung (Figure 1). Aspiration of the fluid in the cavity of the pulmonary cyst revealed* Aspergillus fumigatus* in the exudate, leading to the diagnosis of pulmonary aspergillosis. A cavernostomy was performed and the patient was found to have a cavity filled with purulent attachment and a cauliflower-like fungus ball in the resected specimen. The patient died of respiratory failure 1 month after the cavernostomy.

### 2.2. Case 2 (A 63-Year-Old Male)

*Past History*. Right lower lobectomy with lymph node dissection for right lung cancer at the age of 54. 

*Pathological Findings*. Well-differentiated papillary adenocarcinoma, p-T1bN0M0. 

*Pulmonary Function Test Results*. FVC: 1.65 L, %FVC: 45.8%, FEV_1.0_: 1.62 L, and FEV_1.0%_: 55.1%.

*Comorbidity*. Diabetes mellitus (HbA1c: 6.8%).

**Figure 2:**
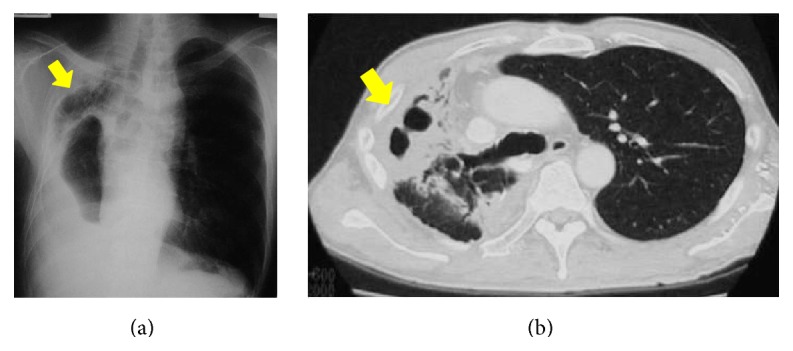
Chest X-ray (a) and chest contrast tomography scan (b) in Case 2. This patient had repeated infections and severe destroyed lung in which almost all the existing lung structures were destroyed.

**Figure 3:**
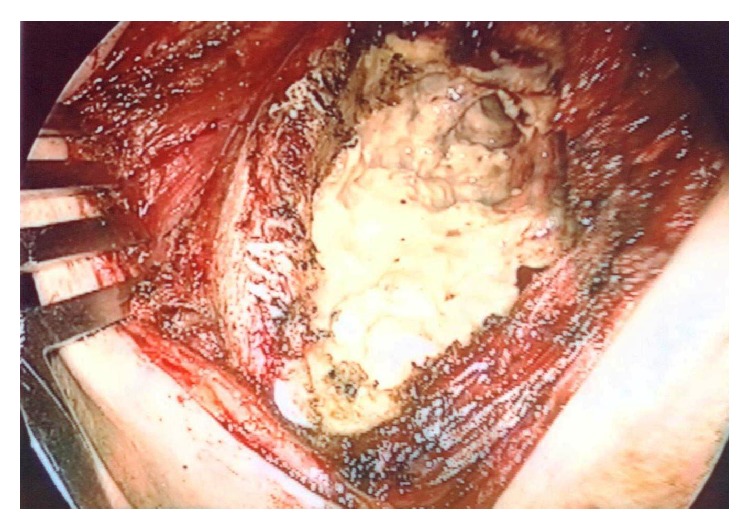
Operative findings in Case 2. Emergency cavernostomy was done for repeated uncontrollable infections.* Aspergillus fumigatus* was detected from the cavity.

*Clinical Course*. Approximately 5 years after resection for lung cancer, the patient developed pneumonia repeatedly and was treated with oral antibiotics. His chest CT showed inflammatory and cystic change immediately below the pleura, but sputum and blood cultures revealed no significant bacteria. At 7 years after his lung resection, repeated bouts of pneumonia became difficult to control with drugs and a chest CT showed severe destroyed lung in which almost all of the existing structure of the lung was destroyed (Figure 2). A cavernostomy was performed and* Aspergillus fumigatus* was identified from the chest cavity, leading to the diagnosis of pulmonary aspergillosis (Figure 3). After the cavernostomy, cleansing of the cavity improved the chronic inflammation and restored the patient's physical strength. A total resection of the residual lung was performed 1 year after the cavernostomy. However, the patient developed a tracheal fistula of about 2 mm in diameter in the membranous portion of the trachea at 14 days after total resection of the residual lung. The patient underwent emergency repair of the fistula with a muscle flap but developed aspergillus-related empyema and died of bleeding from the superior vena cava.

### 2.3. Case 3 (A 72-Year-Old Male)

*Past History*. Left upper lobectomy for left lung cancer at the age of 60.

*Pathological Findings*. Large cell carcinoma, p-T2aN0M0. 

*Pulmonary Function Test Results*. FVC: 1.90 L, %FVC: 62%, FEV_1.0_: 1.28 L, and FEV_1.0%_: 67.36%.

**Figure 4:**
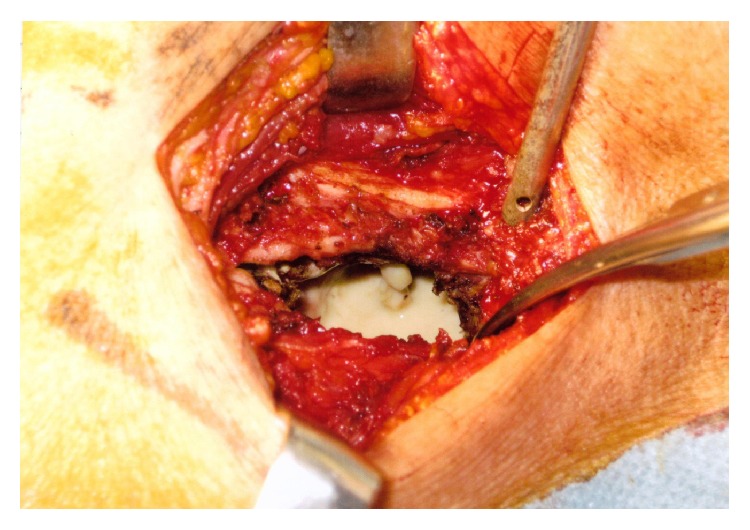
Operative findings in Case 3. The destroyed lung parenchyma and abscess wall were lumped into a mass of fibrous tissue. The cavity contained whitish purulent contents and an irregular-shaped fibrous mass associated with an induration.

*Clinical Course*. The patient lived well without subjective symptoms for 10 years after surgery for lung cancer. Approximately 11 years after surgery, the patient complained of repeated fevers and was treated with oral antibiotics. Sputum and blood cultures did not reveal any significant bacteria. Chest CT at 11 years after surgery showed destroyed lung (left), while CT at 12 years after surgery showed worsening of a left lung abscess and right lung pneumonia. Treatment with antibiotics at that time was not effective and an emergency cavernostomy was performed. There were purulent and tissue-like contents partly associated with induration in the cavity, but no significant bacteria were detected. The intercostal muscle was extremely thin and there appeared to be thickened pleura in the intercostal floor, where the destroyed lung parenchyma and abscess wall were lumped into a mass of fibrous tissue. An incision (1 cm) was made in the wall to reach the cavity. The cavity contained whitish purulent contents and other contents associated with an irregular-shaped fibrous mass and induration. We resected the abscess wall (Figure 4). The postoperative course was uneventful and the patient is being followed at the outpatient clinic.

## 3. Discussion

Pulmonary aspergillosis is one of the most intractable pulmonary diseases, caused by infection and saprophytic growth of* Aspergillus* sp.; a fungus ball forms in existing lung lesions, including the lung cavity after treatment of pulmonary tuberculosis, bronchiectasis, and destroyed lung after surgery for lung cancer [7]. This disease is characterized by a gradual progression, with exacerbations and remissions, and by subsequent extensive destruction of the lung parenchyma associated with symptoms including bloody sputum and hemoptysis, thereby leading to a fatal course [8]. With the introduction of new antifungal drugs, an increased number of patients show treatment effects in response to these medications, including disappearance of the infiltrative shadow and shrinkage of the cavity; however, the rate of complete resolution with medical treatment still remains low because of poor drug penetration to the cavity [9, 10]. Therefore, surgical resection of the affected lung is the primary treatment of choice. According to recent reports of pulmonary resection for pulmonary aspergillosis, the mortality rate is high, ranging from 5.6% to 22.6% [5, 11–13] and suggesting a higher mortality risk when compared with conventional lung resection. Furthermore, many patients with pulmonary aspergillosis are ineligible for lung resection and are forced to have only medical treatment because of advanced age, impaired pulmonary function, or poor nutrition. It is difficult to achieve disappearance of the fungal mass and control symptoms by medical treatment alone, often leading to life-threatening hemoptysis.

We experienced three cases of pulmonary aspergillosis in which cavernostomy was performed because controlling the symptoms by medical treatment was difficult. The characteristics of our patients included an average age of 69 years, mean %FVC of 60.6%, and mean FEV_1.0_ of 1.45 L, suggesting impaired pulmonary function in an older patient population. All of our patients had a previous history of pulmonary resection for lung cancer. The main symptoms observed were pneumonia and bloody sputum, accompanied by prolonged low-grade fever. On imaging studies, destroyed lung was found in all patients.* Aspergillus fumigatus* was detected by aspiration or surgery but not by sputum culture. In our present cases, systemic administration of antifungal drugs was ineffective to control clinical symptoms such as pneumonia and bloody sputum, and pulmonary resection was thought to be impossible due to the patients' poor general condition. We therefore performed cavernostomy, which can be done even in patients with impaired pulmonary function, although this procedure is inferior to pulmonary resection in terms of recurrence.

Cavernostomy, otherwise known as cavernoplasty was established by Nagaishi and Teramatsu as for treatment of a tuberculous cavity in Japan [14]. This is a direct procedure composed of surgical cleansing of the cavity as a source of shedding, followed by primary or secondary closure. These operations attempt to remove the fungal mass and shrink or resolve the cavity. Although these procedures are inferior to pulmonary resection in terms of curability and hemostatic effects, they are frequently used for high-risk patients because of the reduced loss of pulmonary function associated with the surgery. Iuchi et al. reported that cavernostomy in nine patients presenting with repeated massive hemoptysis, aiming at only removing the fungal ball, was markedly effective in terms of successful removal of the mass and relief of hemoptysis in all the patients, although the recurrence rate was 42.9% [15]. However, because cavernostomy is usually performed in patients who are ineligible for pulmonary resection because of poor general condition or impaired pulmonary function (or in patients in whom resection is technically difficult due to dense adhesion caused by previous surgery), the mortality rate is reported to be higher than that seen with pulmonary resection [16].

In all cases, we did not perform ventilation-perfusion scintigraphy. They were post-lung cancer surgery, and the overall status was exacerbated by repeated episodes of fever and bloody sputum. In Case 1, we were not able to control inflammation because medical treatment was ineffective and the appropriate timing for performing a cavernostomy was missed. In this case, immediate cavernostomy should have been performed. In Case 2, the patient's general condition was improved after cavernostomy, but total resection of the residual lung was performed to improve quality of life and complete wound closure. We think that it is necessary to improve the patient's overall status after cavernostomy. Although this operation was performed after waiting for full recovery of the patient's physical strength, we should have considered that the risk of complications after total resection of the residual lung was too high. Further surgery should not have been performed in this patient after the cavernostomy. In particular, pneumonectomy for chronic infectious disease is often technically difficult. When the lung on the affected side becomes a destroyed lung in patients with thoracic empyema and a bronchopleural fistula, extrapleural pneumonectomy is indicated [17, 18]. However, some studies do not recommend extrapleural pneumonectomy because of the high risk of developing postoperative complications [19]; therefore its indication should be carefully determined [18, 20]. In Case 3, we reflected on our experience with Case 1, and we performed an earlier operation. The patient underwent cavernostomy alone because of advanced age and his postoperative course has been uneventful. This is a presentation of retrospective evaluation of these cases. In cases of repeated infection without being able to identify bacteria and in cases where a destroyed lung progresses (as determined by imaging), immediate treatment is important.

## 4. Conclusions

We report three cases of pulmonary aspergillosis associated with destroyed lung developed after pulmonary resection for lung cancer and treated with cavernostomy. Cavernostomy as an emergency procedure was effective in one patient. There are few reports on radical surgery, including additional cavernostomy and total resection of the residual lung. The indication of radical surgery should be carefully determined for each patient after full preoperative investigation of the infecting organisms and evaluation of patient tolerability for the operation.
